# Usage and impact of global biodata resources

**DOI:** 10.1093/bioadv/vbag219

**Published:** 2026-08-08

**Authors:** Carla Cummins, Heidi Imker, Charles E Cook, Guy Cochrane

**Affiliations:** Global Biodata Coalition, EMBL-European Bioinformatics Institute, Wellcome Genome Campus, Hinxton, United Kingdom; University Library, University of Illinois at Urbana Champaign, Urbana, IL, United States; Global Biodata Coalition, EMBL-European Bioinformatics Institute, Wellcome Genome Campus, Hinxton, United Kingdom; Global Biodata Coalition, EMBL-European Bioinformatics Institute, Wellcome Genome Campus, Hinxton, United Kingdom; European Molecular Biology Laboratory, EMBL-European Bioinformatics Institute, Wellcome Genome Campus, Cambridge, United Kingdom

## Abstract

**Motivation:**

Biodata resources constitute a critical, large-scale, and globally distributed infrastructure underpinning life science research, yet their organic growth has hindered efforts to quantify key indicators needed to justify sustainable support, including usage, impact, and interdependencies. Here, we present an updated Global Biodata Coalition inventory alongside a Total Resource Usage (TRU) dataset that integrates this inventory with two complementary literature-derived sources: data citations and informal resource name mentions extracted from full-text articles using a fine-tuned machine learning model. A unified database schema enables cross-resource comparisons, dependency network analyses, and evaluation of resource name distinctiveness.

**Results:**

The combined dataset captures 11.5 million formal and informal references, revealing that most resources are acknowledged informally within article text. Network analysis indicates a densely interconnected ecosystem in which Global Core Biodata Resources function as key providers and integrators, underscoring their foundational role. While full resource names are generally distinctive, widespread use of acronyms limits detectability through text mining. Together, these findings provide robust empirical evidence of a highly utilized and interconnected biodata infrastructure, highlight limitations of single-metric assessments, and underscore the need for multi-dimensional evaluation frameworks and more consistent data citation practices to support informed decision-making and long-term sustainability.

**Availability and implementation:**

The database and analytical code described here are available on https://github.com/globalbiodata.

## 1 Introduction

Biodata resources are life sciences databases that archive research data and/or add value by aggregating and curating those primary research data. There is a large, interconnected ecosystem of biodata resources that collectively form a highly distributed infrastructure widely used by life science researchers globally, most of which provide open access (Martin *et al*. 2021, Ma *et al*. 2023). Biodata resources also provide crucial foundational data for the development of AI models that are dependent on the open data contained within this infrastructure (Burley and Berman 2021, Poveda *et al*. 2026).

This infrastructure has formed organically over the past four decades as individuals, laboratories, and institutions throughout the world have created biodata resources to manage and share research data. Technological developments during this time have resulted in exponential increases in the types and volume of data generated, while advances in storage and computational power have enabled storage and analysis of these new data.

The distributed nature of biodata infrastructure, combined with the independent launch of individual resources, has made it difficult to fully describe or quantify global research activity and impact (National Academies of Sciences, Engineering, and Medicine 2020). Yet such assessments are crucial for establishing a foundation for coordinated and sustainable support. Recent efforts to catalogue databases or measure their impact have provided valuable insights based on a mix of automated and manual curation, community submissions, and/or self assessment tools for individual resources (Sansone *et al.* 2019, Hoyt *et al.* 2022, Insana *et al.* 2023, Ma *et al.* 2023). Each takes a unique approach, focusing on a particular line of evidence (e.g. Ma *et al*.’s article citations) or a challenge to overcome (e.g. Hoyt *et al*.’s interoperability). These examples highlight both the challenge of this work and, as Ma *et al*. noted in their conclusion, the need for multiple factors to be considered to avoid misrepresenting a given resource or research area. In this study, we aim to contribute to this call for a broader approach and integrate multiple forms of evidence to capture a high level view of the resource ecosystem. Our approach is distinguished by its systematic, literature-wide scope in which we apply automated methods across the entirety of the open scientific literature to derive comparable, evidence-based metrics for thousands of resources simultaneously. By comparing resources across shared metrics, we aim to characterise the biodata resource landscape at a system-wide level.

As a first step in describing this infrastructure, the Global Biodata Coalition undertook an inventory of global biodata resources, leveraging a machine learning approach to identify publications describing biodata resources in the open scientific literature. In the first instance in 2022, 3112 individual resources were identified (Imker *et al.* 2023). In addition to a list of biodata resources, the inventory dataset also includes a wealth of metadata describing funders, author locations, and the locations of resources extracted from the identified publications.

Here, we report the successful repetition of the inventory workflow. Additionally, we extended the scope of our analysis to incorporate two additional data types into the dataset. The first comprises Europe PMC’s *data citations*, representing text-mined, accession-based references to datasets from a defined subset of biodata resources. The second captures *resource name mentions*, providing another indicator of how resources are referenced and discussed within and across the literature. This expanded dataset allows us to examine both usage and in-text citation patterns, offering a richer view of how biodata resources are used and acknowledged within the global scientific community. The results provide tangible evidence that these resources operate as an interconnected global infrastructure and highlight their essential role in supporting life science research worldwide.

To support these analyses, we implemented a unified database schema that brings together inventory records, data citation evidence, and resource name mentions within a single structure. Consolidating the underlying workflows in this way enables direct cross-comparison of usage indicators, straightforward construction of dependency networks, and integrated exploration of how resources interact within the broader ecosystem.

## 2 Methods

### 2.1 Working definitions

The definition of *biodata resource* is discussed in depth in Imker *et al.* (2023), and was formed through review of multiple sources and definitions. Broadly speaking, it is defined as “an online source of structured information relating to biological sciences.”

An *inventory publication* is a peer-reviewed publication or preprint authored by a resource’s providers that formally describes that resource, its purpose, and its content (e.g. those commonly published in the *Nucleic Acids Research’*s annual Database Issue). These publications are found in many journals across the life sciences and serve as sources for identifying resources within the inventory, along with structured metadata that aid in describing the resource (e.g. authors, affiliations, funding sources). These publications accumulate formal citations by inclusion within article reference sections to acknowledge the biodata resource itself.

*Resource mention* is defined as the use of a biodata resource name (or pseudonym/alias) within the full text of an article (e.g. “genes were downloaded from Ensembl”). In order to separate ‘true’ mentions from the use of the name as a generic term (e.g. the resource BAR, the Bio-Analytical Resource for Plant Biology, vs the noun “bar”), a machine learning classifier was trained to detect this through context.

*Data citation* is defined as the use of a resource’s identifier or accession number to reference a specific dataset or data accession held by that resource. These citations have been identified for a set of biodata resources with well-established accession/identifier syntaxes as part of Europe PMC’s text mining platform (Ferguson *et al.* 2021).

### 2.2 Updating the inventory of biodata resources

The initial biodata inventory was predicted from Europe PMC articles published between 2011 and 2021 using a fine-tuned version of BioMed-RoBERTa-RCT, a model previously pre-trained on the scientific literature for sequential sentence classification (Imker *et al.* 2023). To update the inventory with years 2022 and 2023, the pipeline was rerun in Google Colab using the published protocol and the Jupyter Notebook initially developed to run the inventory and modified to account for required python package updates in Google Colab (Schackart 2023). That pipeline concatenates newly predicted resources and previously predicted resources, performs a strict deduplication based on name and URL, and then generates an initial inventory that is validated by a human curator to review low-probability predictions and potential duplications based on name or URL. The review was carried out as developed for the original inventory with one additional step to remove spurious special characters introduced by encoding errors. No other changes to the predicted names or other metadata were undertaken. The revised python notebook has been updated in the inventory’s Github repository (https://github.com/globalbiodata) and the revised manual review procedure was updated in Imker and Schackart (2025).

### 2.3 Global Core Biodata Resources

Global Core Biodata Resources (GCBRs) are biodata resources determined to be of fundamental importance to the wider biological and life sciences community and the long term preservation of biological data (https://doi.org/10.5281/zenodo.5845115). GCBRs are selected through a two-step peer-review application process, managed by the Global Biodata Coalition, that evaluates applicant resources based upon a series of quantitative and qualitative indicators. The current set of 52 GCBRs represent a group of high-impact resources expected to show increased connectivity and impact in the global ecosystem of biodata resources. To investigate this empirically, we incorporated GBCRs into our analysis, when appropriate, to compare results for these “core” resources with the entirety of the biodata resources represented in our dataset: see the discussion of resource dependency network analysis in Results.

### 2.4 Design overview

To evaluate the reach and importance of biodata resources within the scientific community, in an open and reproducible manner, we relied on published scientific literature. Article text provides the most reliable source of information about how, and when, scientists are using resources within their work; and the articles themselves are associated with high-quality metadata such as authors, author affiliations, funders, and citations. Centralised literature services such as PubMed and Europe PMC offer robust APIs and bulk downloads for these types of data in a programmatic, well-structured way.

Europe PMC is a large, open data resource of life sciences literature with an API allowing advanced querying and retrieval of detailed metadata and full-text articles. In addition, their powerful text mining service identifies data citations for almost 50 data resources (supplementary Table 1, available at supplementary material  *Bioinformatics Advances* online) and this data is made available to download in bulk (Ferguson *et al*. 2021).

To assess biodata resource usage, we took a three-pronged approach.

1. First, data citation information was imported from Europe PMC. Europe PMC provides bulk CSV files that list individual accessions or identifiers together with the publication IDs in which they appear. These accession–publication pairs were downloaded and then mapped to their corresponding biodata resources within our system. After this import step, each accession record becomes a link from a resource to a publication via one of its identifiers. We refer to this set of resource-publication links as the ‘data citation set’ (DC set).
2. Second, open-access full-text articles were mined for resource name mentions using the names predicted from the updated inventory. A machine-learning model was applied to the processed text to identify candidate mentions based on context. The result here is a set of links from resources to articles, via a matched name or alias. We refer to this set of resource-publication links as the ‘resource mentions set’ (RM set).
3. Third, we identified from the open-access corpus citations to inventory publications that describe biodata resources.

**Figure 1:**
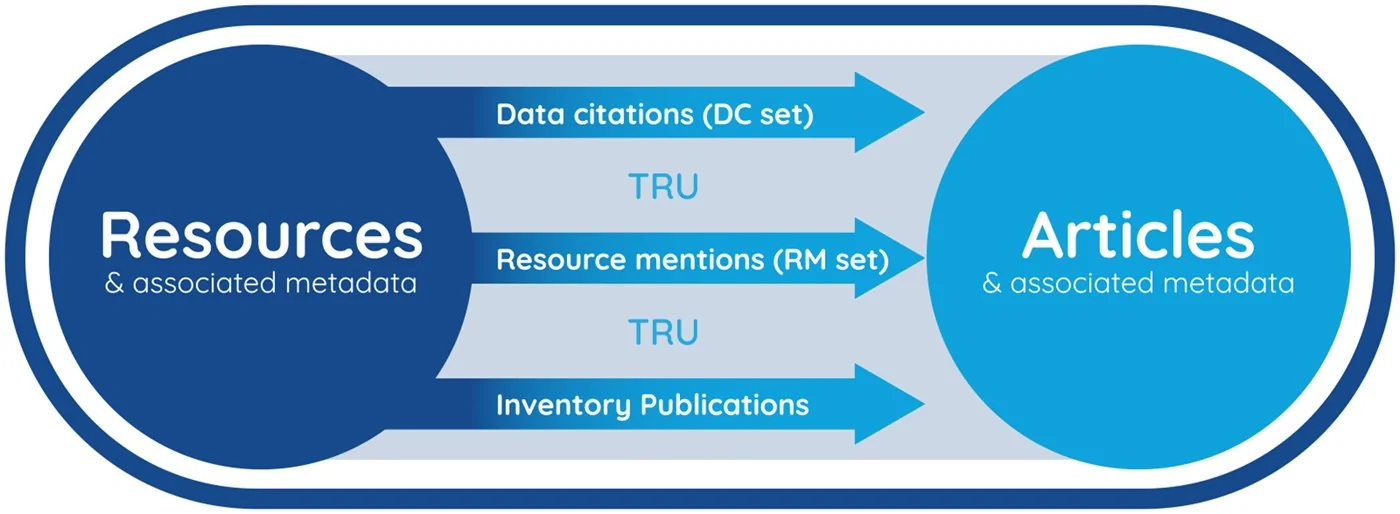
Dataset overview. The basis of this dataset is resources linked to articles, via three complementary link types. Inventory publications: citations to articles describing resources. RM set: articles mentioning resources. DC set: articles citing data from a resource. Highlighted by the light blue box is the “total resource usage” (TRU) set, which is the union of the all three sets.

In all cases, associated articles were also enriched with additional metadata such as funding, affiliations and geographic information. The entire joined set is referred to as the ‘total resource usage set’ or ‘TRU set’ (Fig. 1).

### 2.5 Database & schema

For the purposes of this analysis, a custom MySQL database schema was designed, unifying the outputs of the (recently re-run) inventory workflow (Imker *et al.* 2023), and the TRU set. This uber-schema enables unified exploration and straightforward comparison of resource usage evidence drawn from these three complementary sources.

Publications form the centre of all three analyses, acting as the hub that connects data from different workflows. Extended metadata, such as grants and funding agencies, are captured through dedicated tables, while each workflow or data source has its own tables that link back to the publication table (see Table 1). A detailed schema definition, and schema diagram, is available on GitHub at https://github.com/globalbiodata/gbc-publication-analysis? tab=readme-ov-file#-database-schema-overview.

**Table 1:** Database schema design outline for different data sources.

Data source	Associated schema tables
Inventory	resource, url, connection_status, resource_publication
Data citations	accession, accession_publication
Resource mentions	resource_mentions

For this study, the database was deployed and hosted on Google Cloud Platform.

### 2.6 Data citations set

We imported data citations (DC set) generated by Europe PMC’s text-mining service into the Google Cloud Platform database. As of 2025, there were 52 text-mined accession types with article lists (https://europepmc.org/Annotations#annotation-types-and-information); this list was mapped to 48 GBC inventory resources (Table S1, available at supplementary material  *Bioinformatics Advances* online). We implemented a workflow using the Nextflow workflow manager (Di Tommaso *et al.* 2017, Fig. 2). To maximise throughput while avoiding API rate limitations, bulk data were downloaded from FTP services where available, and only article metadata were queried via the Europe PMC APIs. Metadata fields included title, authors, affiliations, and funding information.

**Figure 2:**
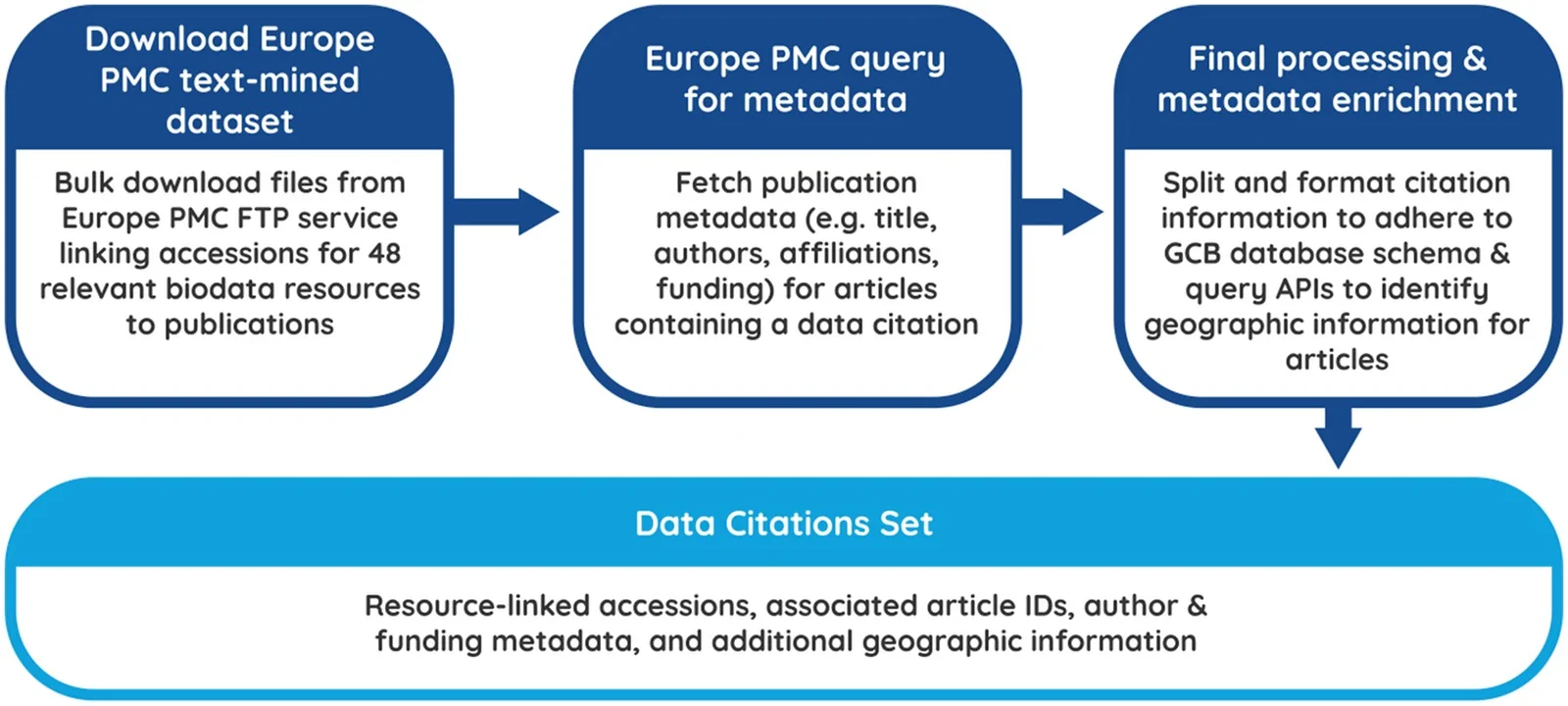
Workflow overview for import of data citations from Europe PMC.

Geographical metadata enrichment was upgraded from that used in the original inventory using a two-step approach. First, affiliation addresses were parsed with the locationtagger Python module (https://github.com/kaushiksoni10/locationtagger), which leverages several Natural Language Processing libraries and geographic databases to identify and extract location names from text. Where no geographic information could be identified by locationtagger, a Google Maps API place search was used as a fallback (https://developers.google.com/maps/documentation/places/web-service).

All data were then normalised to conform to the GCB database schema described above, enabling complex querying and cross-linking in downstream analyses.

### 2.7 Resource mentions set

**Figure 3:**
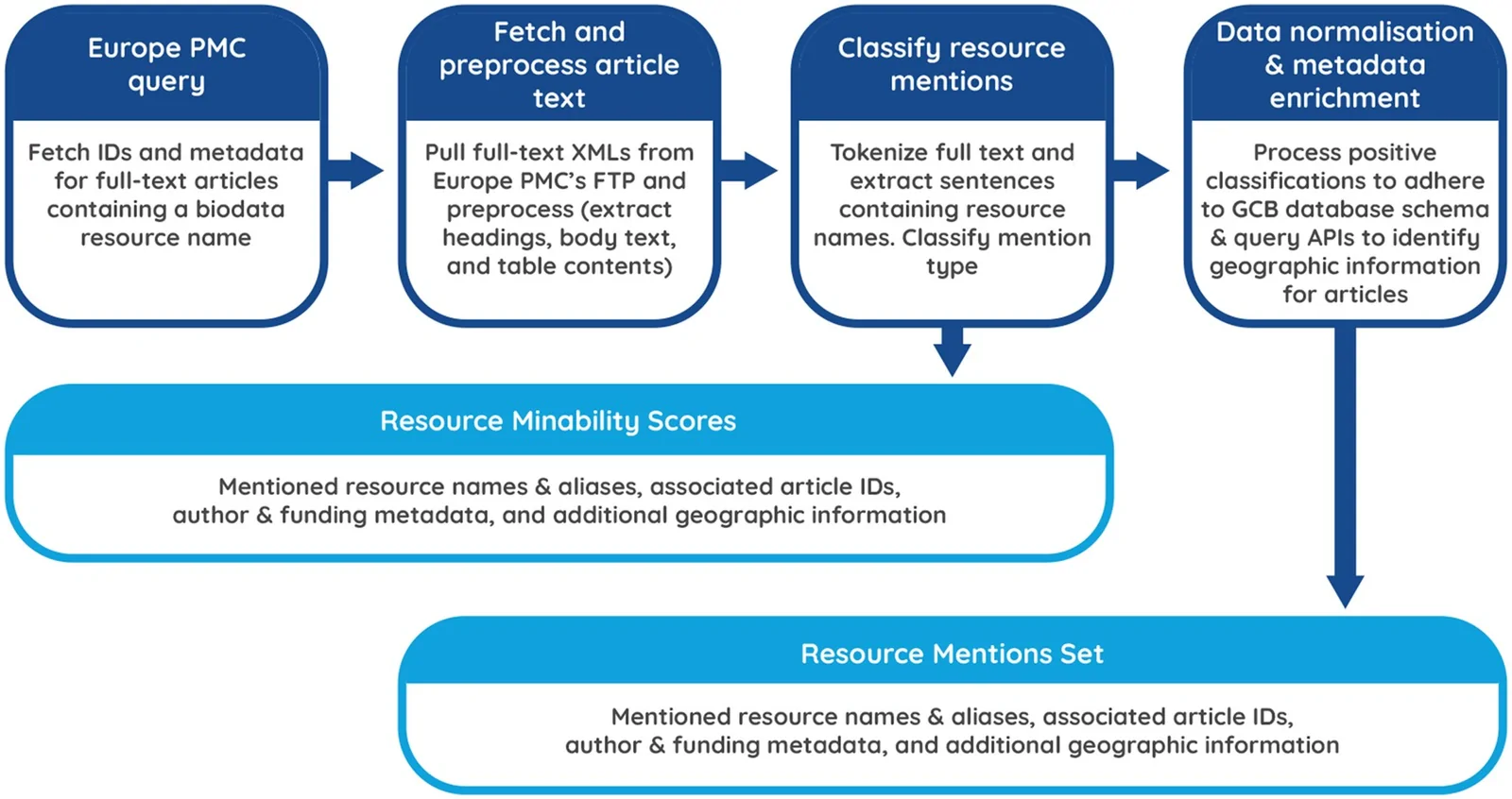
Workflow overview for identification and classification of mentions of biodata resource names in Europe PMC full-text articles.

To generate our RM set, we built a second workflow in Nextflow, and this is available on GitHub at https://github.com/globalbiodata/gbc-mentions-analysis (Fig. 3). Here, data normalisation and geographic metadata enrichment were performed using the same procedures as in the DC set import (see above).

#### 2.7.1 Query and text preparation

As a first-pass filter, basic string-matching searches were performed using the Europe PMC API to identify candidate articles. Each resource, together with any known aliases, was queried against the full open-access article set. Aliases used were largely those predicted by the inventory workflow, with some manually curated aliases were included to ensure GBCRs and renamed resources were not missed. Because a single article could contain mentions of multiple resources, a deduplication step was applied to ensure that each article was fetched and preprocessed only once.

Full-text articles were retrieved separately via the Europe PMC FTP service (https://europepmc.org/ftp/oa/), which provides bundled XML files. Each target article was located within the appropriate bundle, and relevant sections were extracted for analysis. Specifically, the title, abstract, and all text within body tags were collected, while tables were processed to capture all headers and cell text content. The result was a plain-text article with section headers only.

#### 2.7.2 Model fine-tuning

In order to distinguish true mentions of a resource name from the use of the name in a generic context, we needed to train a machine learning classifier. As BERT models have demonstrated strong performance across a wide range of NLP tasks, and several biomedical derivatives have been pre-trained on scientific corpora, they are particularly well-suited to this project. Building on our previous work with the inventory (Imker *et al.* 2023), we employed HuggingFace’s SciBERT model and fine-tuned it for our specific application.

A training set of 1703 sentences containing strings matching resource names was manually curated to include examples where the authors were truly referencing the biodata resource (positively labelled), and examples where the resource name was being used as a generic term (negatively labelled) (Table 2). These were split into 118 tokens (up to 512 characters) for training due to token length restrictions and split into training and test sets.

**Table 2:** Training dataset splits for the mention classification task.

	Train	Test	Total
Positive label	786 (57.2%)	203 (59.0%)	989 (57.5%)
Negative label	588 (42.8%)	141 (41.0%)	729 (42.5%)
Tokens	1,374	344	1,718

Model performance was evaluated on the held-out test set. A true positive (TP) was defined as an article in which a resource name was mentioned in reference to the intended biodata resource and correctly classified as such. A false negative (FN) occurred when such a mention was incorrectly classified. Conversely, a true negative (TN) referred to an article where the resource name appeared in a non-resource context (e.g. common word usage) and was correctly rejected, while a false positive (FP) was an incorrect classification of such a case as a true mention.

From these outcomes, accuracy reflects the overall proportion of correct classifications across all cases. Precision measures the proportion of predicted positives that were in fact true positives, capturing how reliably the model avoids false alarms. Recall measures the proportion of true positives that were correctly identified, capturing how well the model avoids missing relevant mentions. Fine-tuning SciBERT over 4 epochs on our dataset resulted in 96.5% accuracy, 94.7% precision and 98.9% recall.

This fine-tuned model is open-source and available on GitHub at https://github.com/globalbiodata/gbc-mentions-analysis/tree/main/data/models/scibert_resource_classifier.v3.

#### 2.7.3 Article classification task

Plain-text articles were tokenised into sentences for evaluation. As an initial filter, basic string-based matching was performed on each sentence for each resource name to identify candidate mentions. Candidates proceeded to classification using a fine-tuned SciBERT model, where the validity of the mention was assessed.

In addition to recording resource mentions for each article, we tracked the ratio of positive to negative classifications. This provides insight into how specific and reliable a resource name is for text mining, a measure we term the *mineability index*. A mineability index of 1 indicates that *all* basic string matches were confirmed as true mentions, showing the name is highly distinctive and readily mineable. An index of 0 indicates that *none* of the matches were true mentions, meaning the name is ambiguous and poorly suited for text mining.

### 2.8 Resource dependency network construction

To examine interdependencies between biodata resources, we constructed a directed network in which each node represents a resource and each edge (*A → B*) indicates that resource A’s *inventory publication* referenced or used resource B, as identified in the TRU set. In this configuration, edges point from *dependents* to *providers*, capturing inferred flows of collaboration within the ecosystem.

Because the TRU set links publications to resources via mentions or data citations, only those articles identified as *inventory publications* were used to establish these links; that is, publications written by the resource teams describing their own resource. In this way, if an inventory publication refers to another resource, we treat that as an indicator of dependency. However, we note that mentions, in particular, do not necessarily imply functional dependence; rather, they serve as a proxy for interaction or reliance within the community.

The network was analysed in Python using the networkx package. Derived measures included node degree and degree difference (Δ = in-degree–out-degree).

To characterise how widely each resource draws upon or connects to others, we measured *network reachability*. Reachability quantifies the number of distinct nodes that can be reached from a given node within a fixed number of directed steps. In this study, a step represents one edge traversal in the direction of dependency (*A → B*). For example, resources directly referenced in an inventory publication are considered one step away, while those referenced by those resources are two steps away, and so forth. Measuring reachability across multiple step distances provides insight into how far the influence or integration of each resource extends within the network.

## 3 Results

### 3.1 Inventory update results

Prior to the manual review step, the updated pipeline generated an initial inventory with 862 new resources predicated for the years 2022 and 2023. Of these, 235/862 (27.3%) were flagged for a low probably score of < 0.978, with 204/862 (23.7%) flagged for a potential duplicate based on resource name, and 76/862 (8.8%) as a potential duplicate based on URL. After manual review, 182/235 (77.4%) of those with low probability scores were retained and 53/235 (22.5%) removed, with 37 removed due to a partially correct name, 12 for an incorrect name, and 4 for a misclassification. For those flagged as potential duplicates based on name, 135 were annotated for merger, 62 to not be merged, 5 would already be removed due to low probability score, and 2 annotated for a partial merger. Of those flagged as duplicates based on URL, 26 were annotated for merger, 37 to not be merged, and 12 would already be removed due to low probability score. The reviewed inventory, including 8 URLs and 5 names corrected to remove errant character strings, was returned to the pipeline in Google Colab to complete the final processing and augmentation steps that test URL statuses and retrieve additional metadata. This resulted in 661 new resources predicted and validated for 2022 and 2023 and a total inventory, spanning years 2011–2023, of 3773 biodata resources (https://github.com/globalbiodata/inventory_update/blob/master/predictions_final_2024-07-12.csv). The ML-enabled pipeline predicts resources based on the scientific literature, which means biodata resources can be missed via the initial targeted query or if an article explicitly describing the resource does not exist in Europe PMC. Therefore, for completeness of this analysis, 24 resources were manually included in our database, including 11 resources to ensure alignment with the DC set coming from Europe PMC and 13 resources to ensure all GCBRs were included.

### 3.2 TRU set overview

Across the combined Total Resource Usage (TRU) set, we identified 1.7 million publications containing 11.5 million individual references to biodata resources. These included 8.1 million Europe PMC text-mined data citations drawn from 1.3 million publications, covering the 48 distinct resources from the DC set. In total, 3.3 million resource name mentions were detected across 850 k full-text articles, covering 3636 distinct resources and 410 known aliases. Together, these datasets represent the most comprehensive literature-based view of biodata resource usage to date.

The merged TRU dataset forms the basis for all analyses described below. It enables us to explore and infer different aspects of resource usage, from how resources are referenced within the scientific literature, to how they depend on and connect to one another within the broader biodata ecosystem, and the importance of the semantics of resource naming.

### 3.3 Patterns of resource referencing across the literature

In this study, we compared three complementary modes of reference, each reflecting a different level of formality and intent:

1. *Inventory publication citations*: formal citations of the paper(s) describing the resource itself
2. *Data citations (DC set)*: in-text references to specific datasets or accessions from a resource, as identified by Europe PMC’s text-mining pipeline
3. *Resource mentions (RM set)*: informal references to the resource by name within the full text of an article

These modes capture different facets of engagement: citation of an inventory paper indicates formal acknowledgment of the resource, data citations indicate direct reuse of specific datasets or data accessions, and resource mentions signal awareness or conceptual use without explicit linking.

To enable direct comparison across all three reference types, the analyses in this section were restricted in two ways. First, we limited the comparison to the 48 resources present in the DC set, as these are the only resources for which all three reference types are available. Second, because the RM set can only be derived from open-access full-text articles, we restricted all three reference types to this same article pool, ensuring that data citations, resource mentions, and inventory publication citations were compared across the same set of resources and the same set of articles.

**Figure 4:**
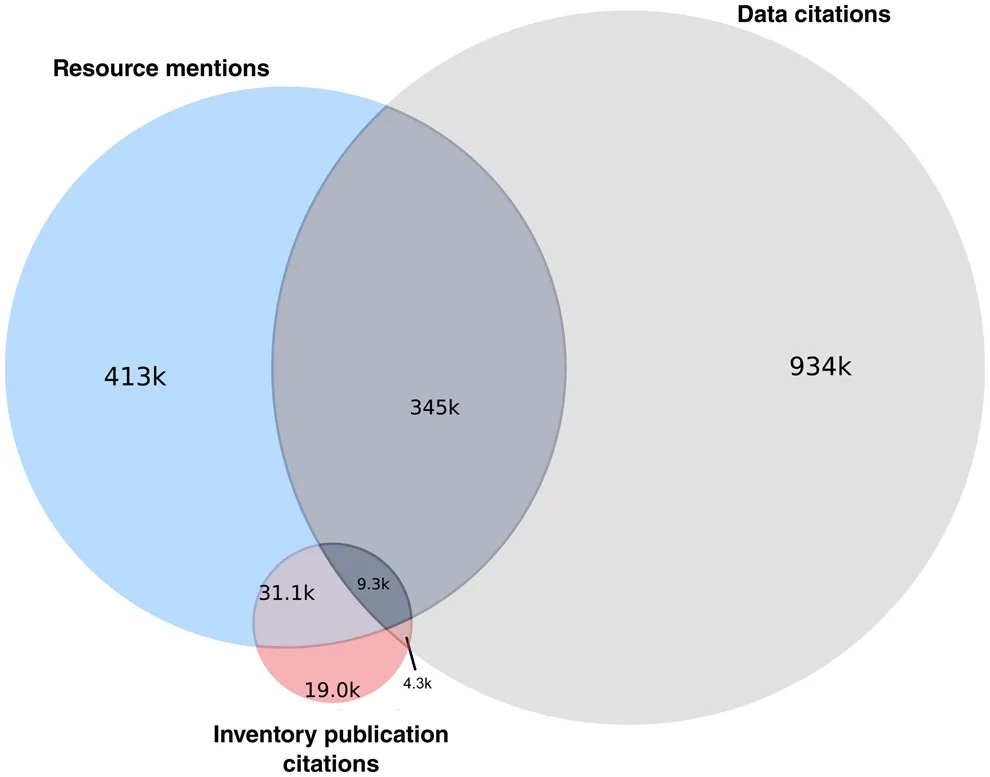
Relative frequencies (*k* = thousands) of different modes of resource referencing in the open access literature across all 48 resources in the Data Citations (DC) set.

**Figure 5:**
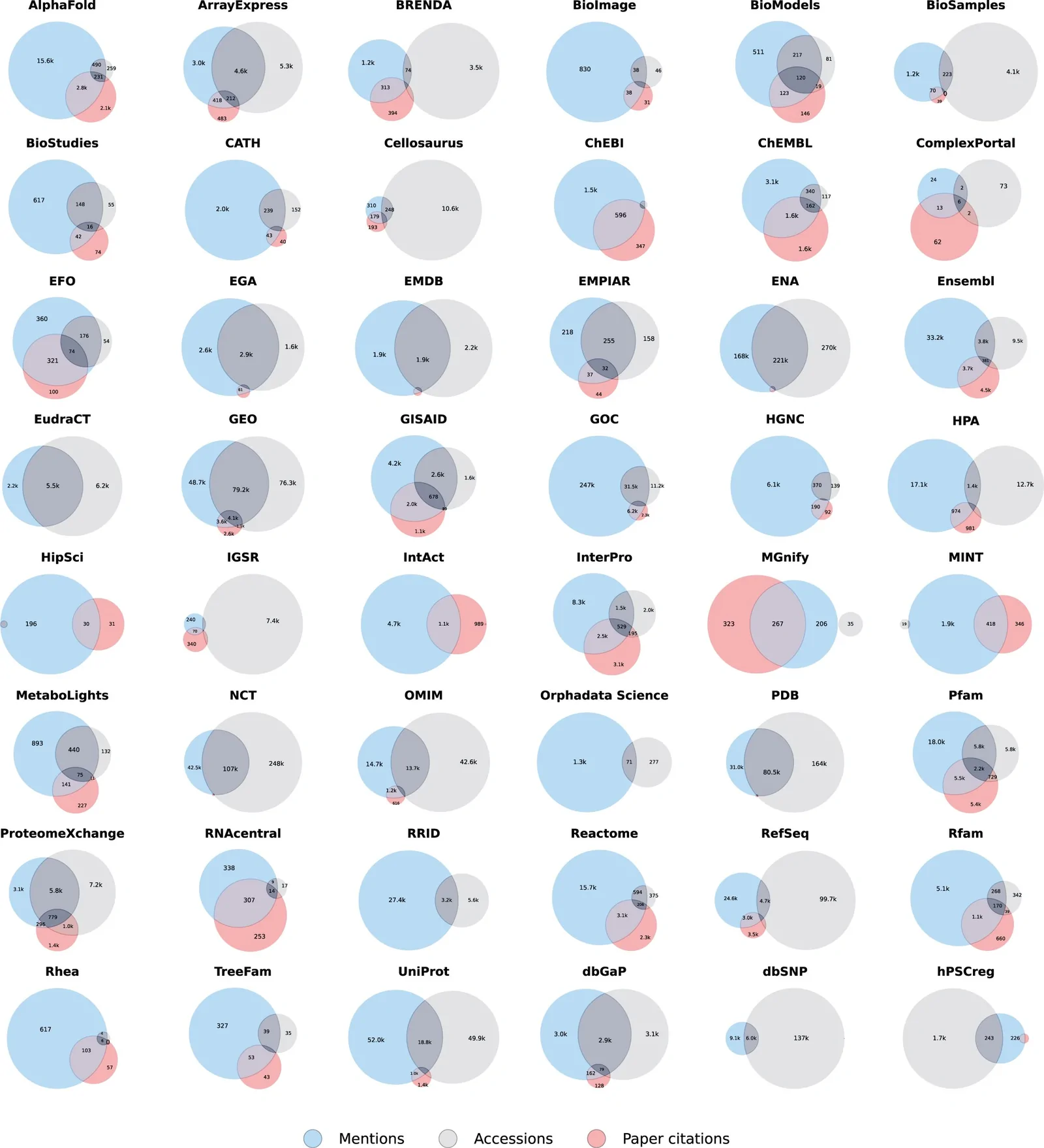
Variation among individual resources in the proportions of citation, accession-based, and name-based referencing. Blue circle represents publications containing resource mentions, grey circle represents publications containing data citations and red circle represents inventory publication citations. See supplementary materials  Table 2, available at supplementary material  *Bioinformatics Advances* online for underlying data. Note that only 17 of the 52 current GCBRs are shown here: this lack of coverage reflects indexing processes outside the direct control of the authors and is by no means an indication that there are no mentions, data citations or literature citations for those GCBRs not included. We hope soon to be able to show full coverage of the GCBRs in these analyses.

At a global level, formal inventory publication citations are vastly outnumbered by both in-text data citations and name mentions (Fig. 4). This highlights that most interactions with biodata resources occur without formal citation, even when resources play a substantive role in data generation or analysis. Patterns also vary widely among individual resources, as illustrated in Fig. 5: some are predominantly referenced through accessions—for example Cellosaurus, while others are mentioned mainly by name—for example HGNC; with very few exhibiting balanced representation across all three categories.

Together, these results emphasise that there is no single standard practice for acknowledging biodata resources or assessing their impact. The diversity of referencing modes underscores the need for more consistent guidance and community norms around how such foundational tools and databases are credited in the literature.

### 3.4 Resource dependency network analysis

Biodata resources form a densely interconnected system, with most resources depending on outputs, identifiers, or standards linked from other resources (Cook *et al.* 2020, Drysdale *et al.* 2020). The directed network formed with our dataset contains 3162 nodes and 27 202 edges, capturing the extensive set of inferred interdependencies identified through the TRU links. Both the mean in-degree and out-degree are 8.6, indicating that, on average, each resource both depends on and supports a similar number of others. This balance suggests a highly integrated and reciprocal infrastructure in which most resources act as both contributors and consumers of shared data.

To explore the extent of mutual reliance within this system, we also examined only those relationships that were bidirectional i.e. where both *A → B* and *B → A* connections existed. When restricted to these reciprocal links, 604 (19.1%) of nodes and 1678 (6.1%) of edges remained, forming 93 connected components. The largest of these components constitutes a core of 320 resources, while the remaining subgraphs comprise small clusters of two to eight nodes, likely representing domain-specific or co-curated partnerships. Based on ELIXIR’s work on core data resources (Drysdale *et al.* 2020), this structure likewise shows a strongly interconnected nucleus of resources surrounded by numerous specialised pairs or small groups. Extrapolating to biodata resources across the broader ecosystem, these results suggest similarly active and sustained data exchange, accompanied by focused and purposeful interoperability.

### 3.5 Degree difference as a measure of contribution

Within this directed network, in-degree represents how many resources rely on a given resource, while out-degree reflects how many others that resource itself depends upon. To summarise this balance, we calculated a degree delta (Δ = in-degree–out-degree) for every node. Positive Δ values identify *providers*: resources that are more frequently relied upon than they rely on others; whereas negative Δ values identify *dependents* that draw more heavily from the wider ecosystem.

**Figure 6:**
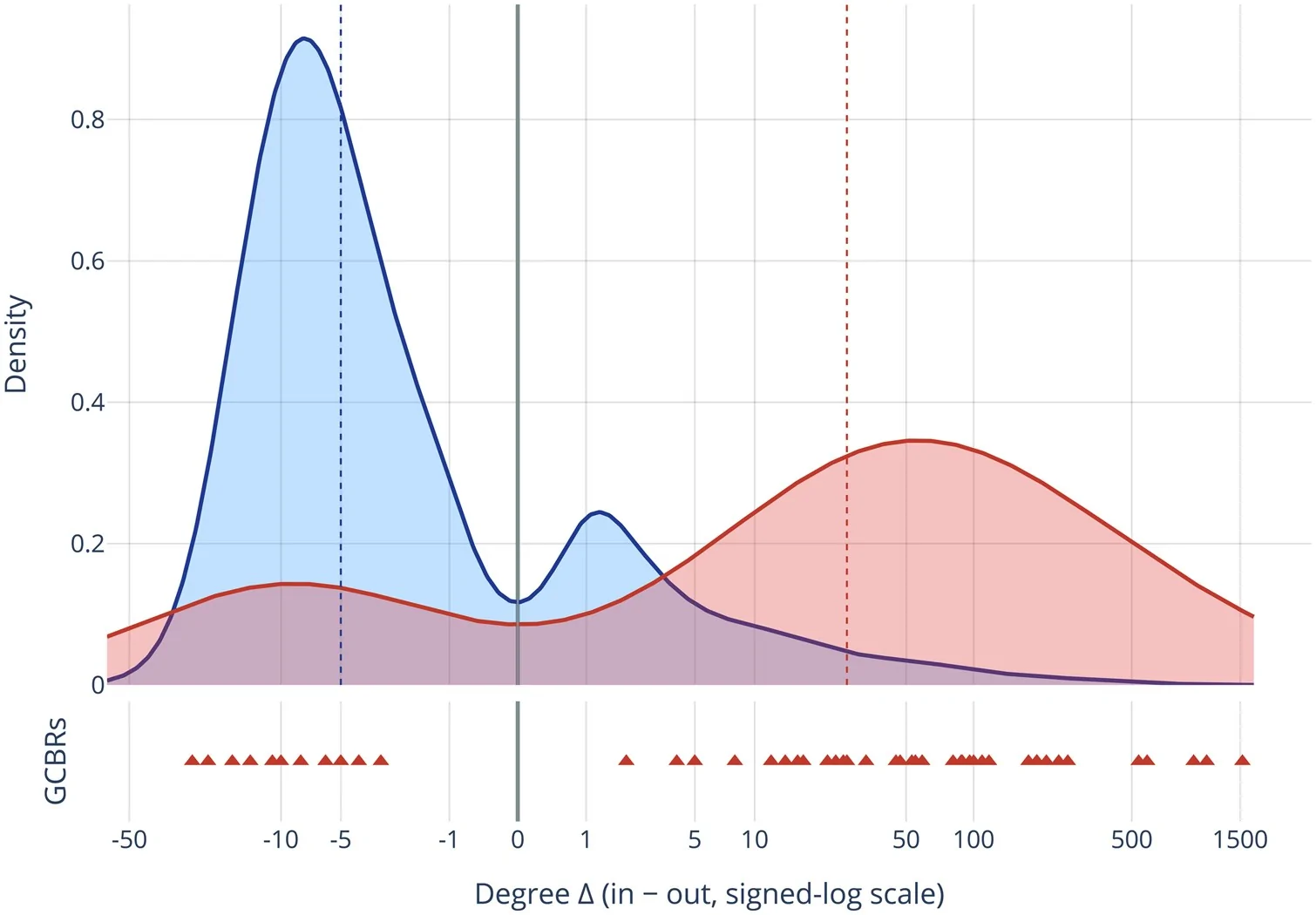
Degree balance (Δ = in-degree–out-degree) for biodata resources. Density of Δ for non-GCBRs (blue, *n* = 3108) and GCBRs (red, *n* = 51), plotted on a signed-log *x*-axis. Dashed lines mark group medians (−5 and +27); triangles below show individual GCBR positions. Most resources cluster just below zero; GCBRs are skewed into the positive tail.

Δ values ranged from –56 to 1534 (mean = 0, median = –5). Of the 3162 resources analysed, 686 (21.7%) had a positive Δ, 2396 (75.8%) a negative Δ, and 80 (2.5%) were neutral. The overall distribution (Fig. 6) shows a strong concentration around small negative values (peak ≈ –5), indicating that most resources are modest net consumers rather than major providers. This pattern is expected in a cooperative ecosystem where specialised databases build upon a smaller number of foundational ones. GCBRs are predominantly located among the positive Δ tail (39 of 52, or 75%), consistent with their role as core, broadly relied-upon nodes in the network.

When compared with mineability results (see Figure 8), a negligible correlation (Pearson’s *r* = –0.038); although nominally significant given the sample size (*P* = 0.032), the effect size is too small to be meaningful. This indicates that a resource’s structural centrality and its textual distinctiveness are independent characteristics.

### 3.6 Network reachability

To highlight how far influence or dependency can extend through the network, we assessed reachability: the number of downstream resources that can be reached from each node over defined step distances. Unlike degree, which reflects only direct neighbours, reachability captures the cascading pathways through which standards, identifiers, and data dependencies propagate.

Reachability increases sharply within the first few steps for most resources, showing how quickly indirect links accumulate even when direct degrees are modest. Many resources that appear locally constrained nevertheless connect indirectly to dozens or hundreds of others through short multi-step chains.

**Figure 7:**
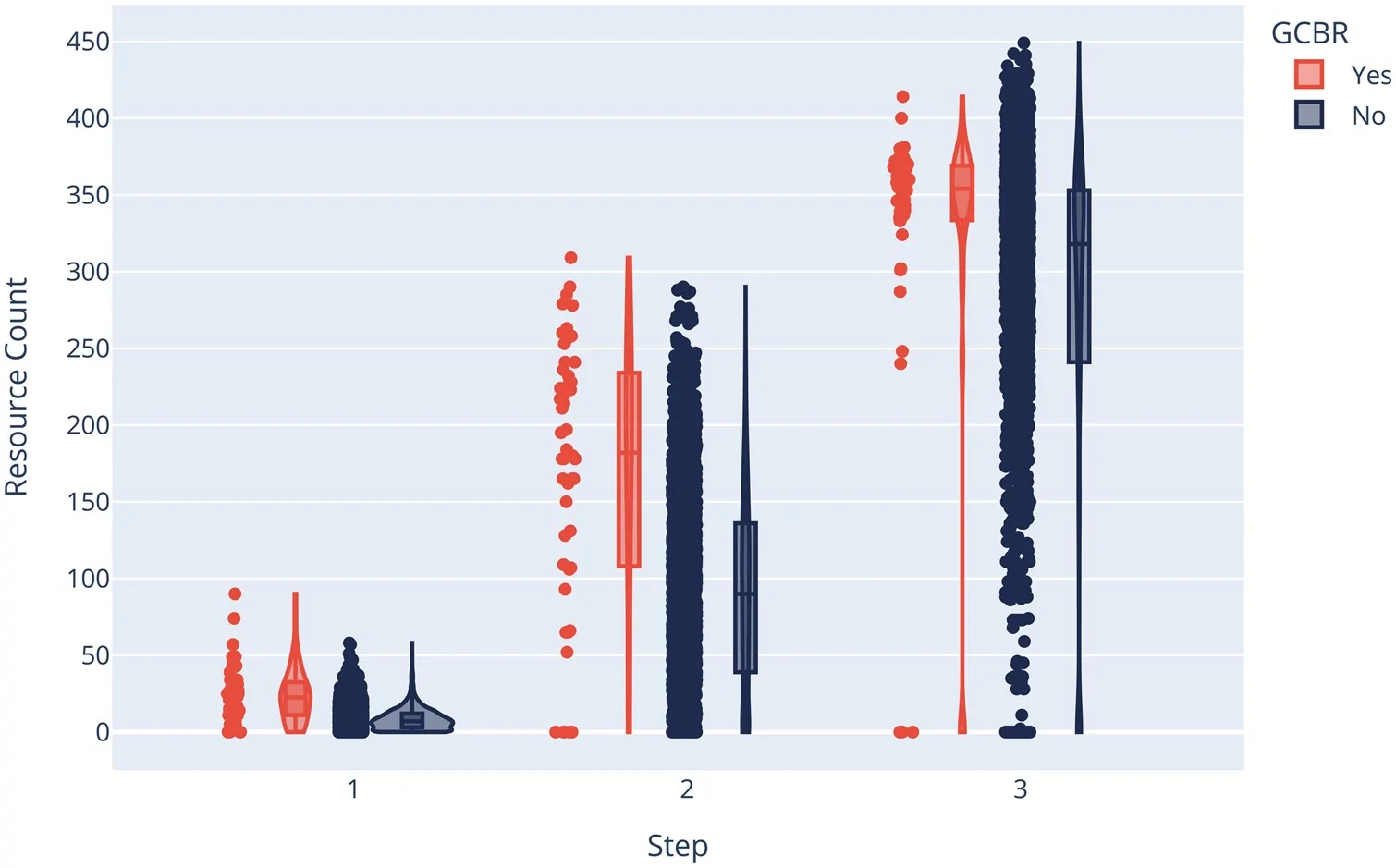
Reachability distributions across step distances. For each step distance from 1 to 3 in the directed dependency network, this plot shows both individual resources as points and the corresponding violin envelopes that summarise their distribution. Each point represents the number of resources reachable from a given node at that step distance. The violins depict the distribution of these points: their width corresponds to the density of observations, and the embedded boxes mark the mean and standard deviation. GCBRs and non-GCBRs are plotted separately at each step to show the differences in their distributions.

The plots in Fig. 7 show several clear structural patterns. As step distance increases, the number of reachable nodes rises for almost all resources. GCBRs maintain higher mean values at every distance and display broader distributions, indicating that they typically access larger portions of the network even at short radii. In contrast, the non-GCBR distribution contains a persistent cluster of near-zero values at every step, marking a set of resources that remain relatively isolated. At step 3, the highest individual reachability values occur in the non-GCBR group rather than the GCBRs. This does not contradict the overall pattern of higher GCBR averages, but it does show that a small number of non-GCBRs reach unexpectedly large parts of the network through longer chains.

Together, these observations highlight the extent of integration across the biodata ecosystem. The steep rise in reachability over the first few steps indicates, at minimum, how quickly resource awareness spreads, which we infer represents interdependency through shared identifiers, standards, and co-referenced datasets. Higher GCBR means underscore their role as major integrators whose outputs underpin connections across the ecosystem. The cluster of near-zero values indicates resources whose influence remains tightly local.

### 3.7 Mineability index distributions

Large-scale data mining and bibliometric analyses currently depend on the ability to identify specific resources by name alone. The *mineability index* provides a measure of how distinctive a resource’s name is as a text string, calculated as the proportion of matched strings that the classifier confirmed as true mentions. An index of 1 reflects perfect specificity, meaning every detected instance referred to the intended resource; an index of 0 indicates complete ambiguity.

**Figure 8:**
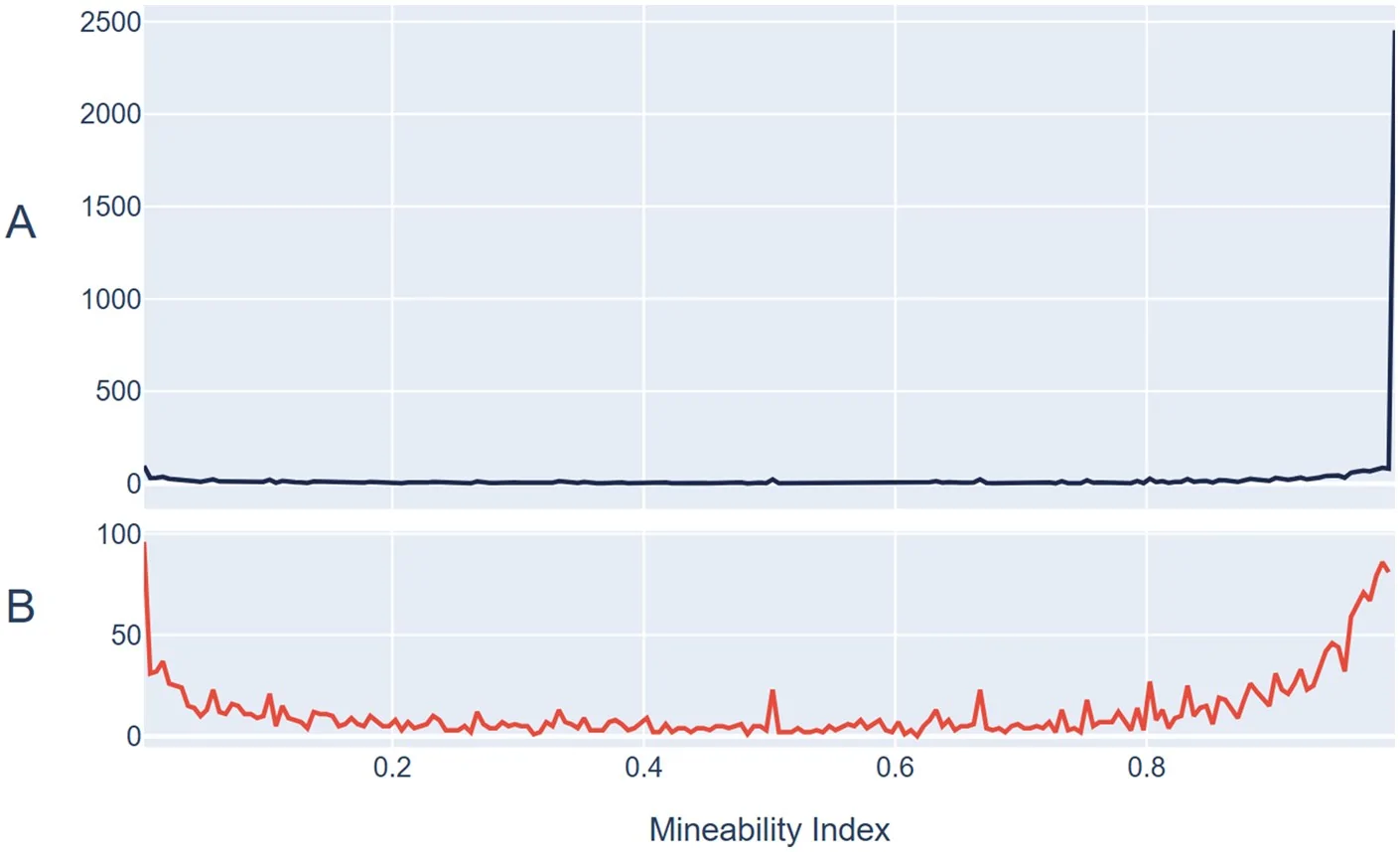
(A) Distribution of mineability indexes for all aliases (0–1). *Y*-axis shows the number of resources at each index point. The sharp increase at the right-hand edge reflects the large number of aliases with an index of 1. This heavy concentration compresses the visual scale and obscures variation among the non-1 values. (B) Distribution with all index = 1 aliases removed, providing clearer resolution of the remaining values. This view highlights a prominent peak at values below 0.05 and a gradual increase in frequency from approximately 0.8 upward.

Across 4092 resource aliases with at least one matched occurrence, 3046 (74.4%) achieved an index above 0.95, showing that most resource names are distinctive and readily recognised (Fig. 8). Only 672 aliases (16.4%) scored below 0.25, often corresponding to short names or common words such as “BAR,” “STRING” or “MicroScope.” Among Global Core Biodata Resources (GCBRs), most aliases scored above 0.9, confirming that these central infrastructure resources are generally referred to in clear and distinctive ways.

While mineability itself does not correlate with scientific impact, it reveals practical challenges in resource discoverability. Many resources maintain multiple aliases or abbreviations that can differ widely in how reliably they are identified in text. For several of the lowest-scoring GCBRs, the poor index values correspond to their shortest aliases (for example, “CIViC” or “RGD”). The longer, descriptive forms (“Clinical Interpretation of Variants in Cancer” and “Rat Genome Database”) are more distinctive but far less frequently used in publications. In the case of CIViC, for instance, 88% of articles use only the acronym despite its lower mineability. This suggests that recognisability in practice is often shaped more by linguistic convenience than by clarity, reinforcing the importance of deliberate, consistent naming: this has been noted in other studies (Imker and Ou 2025). Given the importance of showing impact, choosing names that balance brevity, distinctiveness, and searchability could substantially improve the visibility and traceability of resources in the literature.

These patterns underscore that the discoverability of a resource is determined not only by its scientific reach but also by the linguistic choices underpinning its identity.

## 4. Discussion

Citation behavior is well known to be complex and motivated by cognitive, social, rhetorical, and strategic factors (MacRoberts and MacRoberts 2018, Tahamtan and Bornmann 2019). Within the limits of interpretation, this study provides tangible evidence that biodata resources form a tightly interconnected ecosystem that underpins modern life science research. Our analyses highlight both the depth of these interconnections and the inconsistency with which this critical infrastructure is acknowledged in the literature.

For the 48 resources in the DC subset, formal citation of inventory publications remains uncommon compared with in-text data citations or informal mentions and showed considerable variation across all three types of referencing, consistent with earlier reports (Park *et al.* 2018, Zhao *et al*. 2018). This irregularity, which we suspect is common across all biodata resources, illustrates a fundamental challenge: while researchers routinely use data from established resources, their citation practices often fail to make these dependencies visible. Citation behavior has been a long-standing challenge for the proper acknowledgement of research infrastructure (Mayernik *et al.* 2017), and several studies have shown that data use is dramatically underreported by formal citations (Jonkers *et al.* 2014, Mayo *et al.* 2016, Silvello 2018). In-text citations lack the robust infrastructure that make formal citations a structured, metadata-rich, and interoperable framework that allows wide-spread and readily available tracking and reporting. However, in the absence of formal citations, our study shows that in-text data citations provide a viable alternative to link datasets and publications despite being limited to a relatively small subset of resources where accessions can be reliably mined. At this time, the complexity and heterogeneity of identifier formats, which themselves are not indexed in any centralized way, combined with the computational demands of large-scale extraction, restrict comprehensive coverage.

Name-based references, in contrast, offer the broadest but least standardised view of resource usage. These informal mentions are difficult to quantify, both because of paywalls that restrict text-mining access and also because of linguistic ambiguity. The mineability index results demonstrate how naming choices directly affect discoverability: short or acronymic aliases are much more prone to misclassification than longer, descriptive names (Table 3). This result shows that names play a role in tracking use and, ultimately, impact of biodata resources. Some resources that are presumably widely used, including several GCBRs, have low mineability despite their central role in the research ecosystem. This disconnect between scientific importance and textual recognisability underscores the need for more deliberate, consistent naming strategies and for metadata infrastructures that associate resource aliases unambiguously.

**Table 3:** Distribution of mineability indexes, grouped by range, showing how alias character length correlates with discoverability and distinctiveness.

Index range	Total aliases	GCBR aliases	Mean alias character length
**0.0–0.1**	444	3	6.7 (std dev 7.4)
**0.1–0.2**	171	2	5.9 (std dev 7.4)
**0.2–0.3**	110	2	6.6 (std dev 10.5)
**0.3–0.4**	111	4	8.3 (std dev 9.2)
**0.4–0.5**	101	8	11.8 (std dev 12.3)
**0.5–0.6**	79	0	8.2 (std dev 9.1)
**0.6–0.7**	111	4	10.3 (str dev 10.5)
**0.7–0.8**	152	3	11.4 (std dev 11.4)
**0.8–0.9**	294	7	9.7 (std dev 9.4)
**0.9–<1.0**	1012	29	9.4 (std dev 7.1)
**1.0**	2317	37	19.7 (std dev 14.6)

Each value falls into exactly one bin; lower bounds are exclusive except at 0, and upper bounds inclusive.

The dependency network analysis provides a complementary perspective, corroborating prior evidence that defined sets of biodata resources, for example the resources managed at the EMBL European Bioinformatics Institute and the ELIXIR Core Data Resources are robustly inter-connected, both with each other, and with other biodata resources globally (Cook *et al.* 2020, Drysdale *et al.* 2020). Our work extends these examples using defined sets of biodata resources to demonstrate interconnectedness across the entire global infrastructure as a whole. From the exchange of citations and mentions, we infer that most act simultaneously as providers and consumers, forming a cooperative infrastructure in which information flows bidirectionally. A substantial subset of resources engage in fully reciprocal exchanges of citations or mentions, forming a tightly connected core surrounded by smaller, domain-specific clusters and likely reflecting the specialised collaborations underpinning the ecosystem.

The set of Global Core Biodata Resources as a group show more and deeper connections to other biodata resources globally (Fig. 7). This is as expected since GCBRs are selected because they are, by definition, core resources within the global infrastructure, and the work here provides another line of evidence that these resources serve as integrators that propagate interoperability across domains. Other resources in the network (i.e., non-GCBRs) are on average less connected within the global infrastructure, again as expected. However, a small number of non-GCBR resources are more highly connected than the set of GCBRs, indicating, as was already suspected, that some of these resources might qualify as GCBRs in future selection rounds.

Recognising the duality in the way data resource dependencies can be tracked is essential for both funders and policymakers. Quantitative indicators based solely on formal citations underestimate the influence of foundational infrastructure. A combined approach that integrates network metrics with text-mined usage patterns provides a more realistic picture of how data resources support global research. The mineability analysis, in contrast, highlights the limitations of such metrics by showing how inconsistent naming and linguistic ambiguity can obscure true patterns of use. Strengthening resource acknowledgement will require clearer community guidance–and uptake–on citation practices and technical improvements that make resources easier to identify automatically.

Training data for this study focused on the identification of true positives for resource mentions, a challenging distinction in and of itself. These verified links were then used to explore patterns of co-citation and propagation. Future training could focus on the purpose of citations or mentions within the text, providing deeper evidence of the specific nature of interactions between resources across the entire global community Our work provides ample evidence that such a study is both feasible and well worth undertaking, and that a multi-pronged approach that takes into account the many ways in which a resource is acknowledged is likely to be fruitful for developing an increasingly more comprehensive and compelling view of the impact of this distributed, yet crucial, infrastructure.

## 5. Conclusion

By leveraging the GBC biodata inventory and integrating multiple metrics to include formal article citations as well as informal references through mentions and accessions, we provide robust evidence of the scale, reach, and interconnectedness of global biodata resources. Our results augment and extend prior studies that have focused on individual resources or single metrics. Such studies provide invaluable insights about which metrics are most promising, and we build on these by testing whether those signals hold across a wider variety of resources and at scale. In addition to continuing to evaluate the global impact of biodata resources, this allowed us to test these metrics’ generalizability, as well as surface the limitations and challenges. In this study we report extensive evidence of biodata resource use through both formal and, especially, different forms of informal citations. Importantly, we also show that the relative proportions vary considerably between individual resources. These findings clearly demonstrate that the reliance on any single metric can obscure the true value of individual resources and the aggregate value of biodata infrastructure worldwide. This work highlights the importance of multi-dimensional evaluation frameworks and the continued need to improve data citation practices, which remain inconsistent despite years of advocacy. By providing robust empirical evidence, this study helps ground stakeholders’ decisions and efforts as we collectively work toward building a more sustainable ecosystem.

Additionally, biodata resources have already proven enormously valuable for the development of AI models capable of contributing to scientific advances, and there are on-going efforts to define “AI readiness” for data (e.g. Sansone *et al*. 2022, Clark *et al*. 2024, Hiniduma *et al*. 2025). To capitalize on vast, but highly distributed, troves of existing data, the ability to identify and prioritize resources with the potential to contribute valuable data that is not yet AI-ready is essential. In this light, our analysis highlights both the strengths and limitations of text-based methods for automated discovery and retrieval, which have become increasingly central in the era of AI, for the biodata resources themselves. These approaches offer substantial power to quickly and reliably identify, link, and analyze resources at scale, particularly when accompanied by contextual information, consistent naming practices, and standardised identifier schemas. At the same time, our findings highlight clear opportunities for improvement in each of these areas. Continued progress will enhance the effectiveness of computationally-driven methods for mapping and understanding the biodata ecosystem, resulting in greater comprehensiveness and efficiency on multiple fronts.

## Supplementary Material

vbag219_Supplementary_Data

## Data Availability

All code and documentation is available on GitHub. The Global Biodata Coalition’s repository: https://github.com/globalbiodata. The complete database contents are available as a compressed MySQL file at: https://doi.org/10.5281/zenodo.17672170.
